# Gelatin Tight-Coated Poly(lactide-*co*-glycolide) Scaffold Incorporating rhBMP-2 for Bone Tissue Engineering

**DOI:** 10.3390/ma8031009

**Published:** 2015-03-10

**Authors:** Juan Wang, Dongsong Li, Tianyi Li, Jianxun Ding, Jianguo Liu, Baosheng Li, Xuesi Chen

**Affiliations:** 1 Key Laboratory of Cancer Prevention and Therapy, Tianjin Medical University Cancer Institute and Hospital, Tianjin 300070, China; E-Mail: wangjtju@gmail.com; 2 Department of Radiation Oncology, Shandong Cancer Hospital, Shandong Academy of Medical Sciences, Jinan 250117, China; 3 Department of Orthopaedic Surgery, the First Hospital of Jilin University, Changchun 130021, China; E-Mail: lidsjlu@gmail.com; 4 Orthopedics Dept. 2, Heilongjiang Provincial Corps Hospital of Chinese People’s Armed Police Forces, Harbin 150076, China; E-Mail: lity1978@gmail.com; 5 Key Laboratory of Polymer Ecomaterials, Changchun Institute of Applied Chemistry, Chinese Academy of Sciences, Changchun 130022, China; E-Mails: jxding@ciac.ac.cn (J.D.); xschen@ciac.ac.cn (X.C.)

**Keywords:** biomaterials, bone tissue engineering, gelatin, surface coating, recombinant human bone morphogenic protein-2

## Abstract

Surface coating is the simplest surface modification. However, bioactive molecules can not spread well on the commonly used polylactone-type skeletons; thus, the surface coatings of biomolecules are typically unstable due to the weak interaction between the polymer and the bioactive molecules. In this study, a special type of poly(lactide-*co*-glycolide) (PLGA)-based scaffold with a loosened skeleton was fabricated by phase separation, which allowed gelatin molecules to more readily diffuse throughout the structure. In this application, gelatin modified both the internal substrate and external surface. After cross-linking with glutaraldehyde, the surface layer gelatin was tightly bound to the diffused gelatin, thereby preventing the surface layer gelatin coating from falling off within 14 days. After gelatin modification, PLGA scaffold demonstrated enhanced hydrophilicity and improved mechanical properties (*i.e.*, increased compression strength and elastic modulus) in dry and wet states. Furthermore, a sustained release profile of recombinant human bone morphogenetic protein-2 (rhBMP-2) was achieved in the coated scaffold. The coated scaffold also supported the *in vitro* attachment, proliferation, and osteogenesis of rabbit bone mesenchymal stem cells (BMSCs), indicating the bioactivity of rhBMP-2. These results collectively demonstrate that the cross-linked-gelatin-coated porous PLGA scaffold incorporating bioactive molecules is a promising candidate for bone tissue regeneration.

## 1. Introduction

Clinical studies have shown that the adequate implantation of filling materials for treating critical bone defects resulting from traumatic injuries or tumor resections can facilitate bone regeneration [1]. Autografting is the current conventional treatment for bone defects. However, the process involves a number of drawbacks, including an additional surgical harvesting procedure, and the risk of infection or donor-site morbidity [2]. Allograft materials, such as demineralized bone matrix, also present a risk of immunologic rejection or disease transmission. These factors impede the wide application of the autografting process [3].

The biodegradable polymeric scaffolds for bone reconstruction have received significant attention because of their ability to provide a spatially and temporally appropriate environment for new bone tissue growth [4,5]. Polylactone-type biodegradable polymers, such as poly(L-lactide) (PLA), polyglycolide (PGA), and their copolymer, poly(lactide-*co*-glycolide) (PLGA), are the Food and Drug Administration (FDA)-approved matrices of scaffolds due to their low immunogenicity, non-toxicity, and adjustable degradation rate [6]. However, these polymeric scaffolds impede cell attachment and penetration due to the lack of cell anchoring sites, and they have poor hydrophilicity and low surface energy [7].

Many approaches have been developed to circumvent these drawbacks, including bulk modification and surface modification [8,9]. Bulk modification introduces functional groups to the polymer chain while it may also alter the mechanical and biodegradable properties of the material, which may lead to the undesirable results [10,11]. Surface modification allows the binding of functional groups or bioactive molecules, such as collagen, gelatin, RGD, and hydroxyapatite, onto the surfaces of scaffolds. A few of more complicated surface modifications require chemical reactions [12,13]. However, due to the lack of functional groups on the polymer backbone, it is difficult to modify the surface properties by conventional chemical methods [13]. Surface coating is a simple and general approach for surface modification. However, the bioactive molecules typically do not spread well on polylactone-type skeletons. Additionally, the interactions between the polymer backbone and the bioactive molecules are weak, which leads to an unstable surface coating [11]. To increase the coating efficiency of a polymeric scaffold, a loose polymer backbone structure can be designed, so that the bioactive molecules can easily diffuse across the skeleton and simultaneously increase the adhesiveness for other bioactive molecules to prevent coating loss.

Dunn *et al.* [14] first introduced an *in situ*-formed implant based on phase separation triggered by a solvent/non-solvent exchange, a process that has been applied to tissue engineering and drug delivery for many years [15,16,17]. A water-insoluble biodegradable polymer is first dissolved in an organic solvent that is miscible or partially miscible with water. Following immersion into an aqueous medium, phase separation occurs as the solvent diffuses toward the surrounding aqueous environment, while water penetrates into the organic phase. This process results in polymer precipitation and the formation of implants with a loosened skeleton. For example, Ellis *et al.* [16] produced PLGA flat sheet membranes with a finger-like structure using 1,4-dioxane and 1-methyl-2-pyrrolidinone (NMP) as solvents and water as the non-solvent. Porous structures are expected to form in the high mutual affinity-NMP-water medium. Oh *et al.* [17] fabricated the hydrophilic porous PLGA tubes using a modified immersion precipitation method and showed that the tubes were highly effective for the permeation of bovine serum albumin (BSA).

In this study, an immersion separation method was used to design and fabricate a loosened scaffold with skeletal structure and subsequently carried out the surface modifications by immersing the scaffold in a gelatin solution. Gelatin is derived from high molecular weight collagen by breaking the natural triple-helix structure of collagen into single-stranded molecules; it has been used in many aspects of tissue engineering because of its biocompatibility and ease of gelation [18]. Due to the loosened structure of the biopolymer skeleton, gelatin can easily spread across and over the scaffold surface. After a simple cross-linking procedure, gelatin binds tightly throughout the structure, thereby preventing the surface-coating gelatin from easily falling off. Moreover, gelatin is also an ideal carrier for protein delivery [19,20]. In a previous study, the unique release profile of recombinant human bone morphogenetic protein-2 (rhBMP-2) was assessed in gelatin-coated 3D scaffolds, showing first a transient burst and then sustained release profile [20]. Along similar lines, in this study, rhBMP-2 was incorporated by physically entrapment in a gelatin gel. This multifunctional scaffold composed of a PLGA skeleton, gelatin coating, and rhBMP-2 was further evaluated for cell adhesion, proliferation, and differentiation properties.

## 2. Results and Discussion

### 2.1. Scaffold Characterizations

#### 2.1.1. Microstructure Detections of 3D Porous Scaffolds

The biocompatibility with cells and tissues of a material surface is determined by the interaction between the cells and the surface of material [10]. Due to their hydrophobicity, PLGA scaffolds are not able to well support cell adhesion and growth. When coated with gelatin, the scaffolds gain the hydrophilic property and cell-recognizable moiety [21,22,23]. However, the gelatin solution is not able to infiltrate deeply enough into the macropores of the polymeric substrate to form a stable composite; additionally, the gelatin layer on the exterior surface is unstable due to the insufficient adhesion force between the gelatin and the polymer material [23].

**Figure 1:**
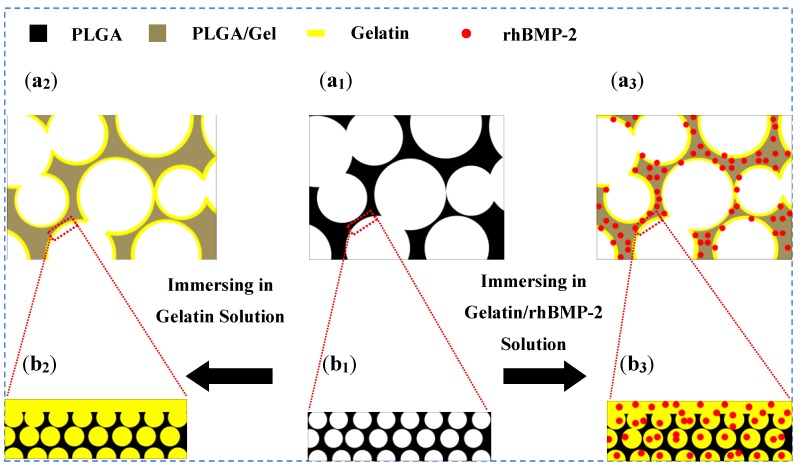
Schematic diagram of surface coating on PLGA scaffold (**a_1_**,**b_1_**), and the PLGA scaffolds coated with gelatin (PLGA/Gel; **a_2_**,**b_2_**) and gelatin/rhBMP-2 mixture (PLGA/Gel/rhBMP-2; **a_3_**,**b_3_**).

In this study, as depicted in Figure 1, a PLGA-based scaffold with a loosened skeleton was fabricated by phase separation triggered by a solvent/non-solvent exchange (Figure 1a_1_,b_1_). The gelatin solution was able to easily penetrate into the loosened skeleton, and the surface gelatin coating was stabilized due to the cross-linking bonds with the glutaraldehyde-modified gelatin secured within the PLGA skeleton (Figure 1a_2_,b_2_). No functional group of PLGA was involved in the surface modification. In contrast with the methods that use modifying groups in copolymerization, this technique maintains the bulk properties of the materials. Furthermore, growth factors like rhBMP-2 could be easily sealed in the scaffold for controlled release by immersing the PLGA scaffold in gelatin solution supplemented with rhBMP-2 (Figure 1a_3_,b_3_).

Phase separation triggered by solvent/non-solvent exchange has previously been applied to fabricate porous nerve guide conduits and bone graft substitutes [15,16,17,24,25,26,27]. The asymmetrical porous structure is formed during the preparation of the biomaterials. Smaller pores are formed at the solvent/non-solvent contact side, when the polymer precipitates due to a higher initial polymer concentration as the non-solvent slowly diffused into the PLGA substrate. The larger pores are formed from the precipitation of the polymer at a lower polymer concentration relative to the initial contact side [17].

After soaking in gelatin and gelatin/rhBMP-2 solutions, the physicochemical properties of the scaffolds were determined and are summarized in Table 1. The PLGA/Gel and PLGA/Gel/rhBMP-2 scaffolds had gelatin contents of 13.8 ± 3.7 and 14.5 ± 4.1 wt%, respectively. After coating PLGA scaffold with gelatin or gelatin/rhBMP-2, the porosity of scaffold slightly decreased from 89.1% ± 8.3% to 74.7% ± 10.1% or 75.5% ± 7.9%, corresponding to a pore diameter decrease from 243.6 ± 72.8 to 219.8 ± 97.5 or 214.4 ± 106.3 μm, respectively. These results indicated that gelatin was successfully incorporated into the PLGA scaffold. More importantly, after gelatin-coating, the PLGA skeleton still retained properties compatible with bone regeneration, *i.e.*, a porosity of 30%–90% and a pore size of 100–1000 μm, ranges that were considered ideal for the growth of bone tissue inside an implant.

**Table 1:** Physical properties of scaffolds.

Scaffold	Gelatin content (wt%)	Porosity (%)	Pore diameter (μm)
PLGA	0	89.1 ± 8.3	243.6 ± 72.8
PLGA/Gel	13.8 ± 3.7	74.7 ± 10.1	219.8 ± 97.5
PLGA/Gel/rhBMP-2	14.5 ± 4.1	75.5 ± 7.9	214.4 ± 106.3

**Figure 2:**
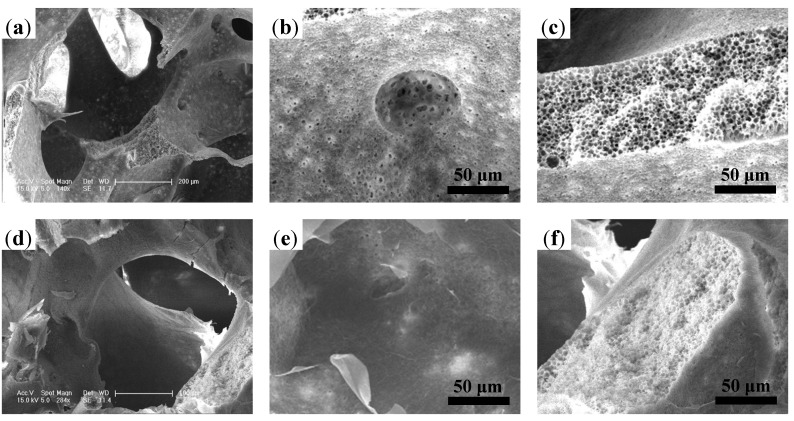
SEM microimages of internal structures for (**a**–**c**) porous PLGA scaffold and (**d**–**f**) PLGA scaffold with gelatin coating.

The microstructures of the three-dimensional (3D) porous PLGA, PLGA/Gel, and PLGA/Gel/rhBMP-2 scaffolds fabricated *via* the phase separation/particulate leaching method were observed by SEM and microscopy (Figure 2). The PLGA microstructure had well-interconnected macropores (Figure 2a), which were ideally suited for cell infiltration. As shown in Figure 2b,c, the skeleton had a honeycomb-like structure composed of microvoids with diameters of 2–4 μm, and the PLGA surface contained microscale channels, to which the internal macropores and microvoids in the skeleton were connected. The observed architecture was very favorable for the movement of proteins. Oh *et al.* [17] demonstrated that macromolecules can easily flow into the microvoids.

Surface modifications were carried out by submerging the PLGA scaffolds in 1% gelatin solutions. Due to the hydrophobicity of the PLGA-based material, a negative pressure was applied to facilitate the infiltration of gelatin solution into the scaffold. After cross-linking with glutaraldehyde, the gelatin was coated on the surface and stored in the microvoids, as illustrated in Figure 1a_2_,b_2_. Figure 2d shows the microstructure of the PLGA/Gel scaffold. The addition of gelatin did not affect the interconnections between the macropores. This observation was consistent with those of previous studies, wherein poly(ε-caprolactone) scaffolds still possessed satisfactory interconnectivity after coating with a 5% gelatin solution [20]. The surface topography of the gelatin-coated PLGA/Gel scaffold is shown in Figure 2e. A thin gelatin layer was tightly adhered to the wall, and the channels almost disappeared. The PLGA substrate after gelatin modification became more compacted (Figure 2f), indicating that the honeycomb-like structure in the substrate was partially filled with gelatin.

**Figure 3:**
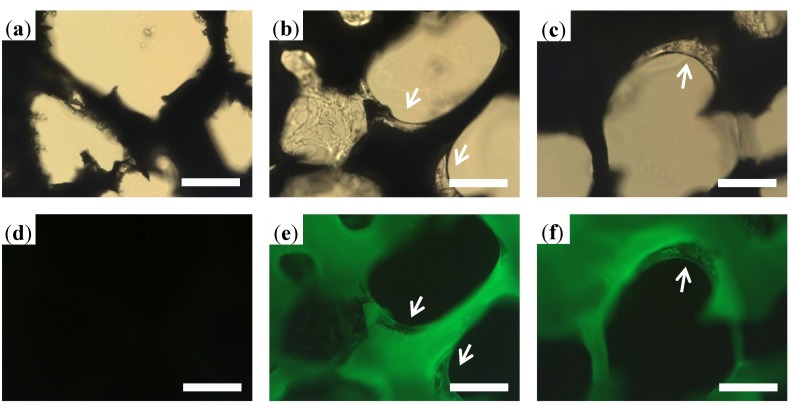
Fluorescence microscopic images of internal structures of porous PLGA scaffold (**a**,**d**), and PLGA scaffolds with gelatin (**b**,**e**) and gelatin/rhBMP-2 coating (**c**,**f**). Scale bar = 100 μm.

To further confirm the gelatin distribution in the substrate, the frozen sections of the PLGA, PLGA/FITC-Gel, and PLGA/FITC-Gel/rhBMP-2 scaffolds were observed under optical and fluorescence microscope. The inner structures of the scaffolds are shown in Figure 3, from which the gelatin coatings are clearly observed in both the PLGA/FITC-Gel and PLGA/FITC-Gel/rhBMP-2 scaffolds, as indicated by white arrows in Figure 3b,c. As shown in Figure 3e–f, the fluorescence images of the PLGA/FITC-Gel and PLGA/FITC-Gel/rhBMP-2 scaffolds showed that the gelatin surface and skeleton both emitted at high intensities, suggesting that the FITC-gelatin had penetrated into the substrate. Due to the cross-linking of gelatin within the substrate, the gelatin coating was firmly secured on the surface.

**Figure 4:**
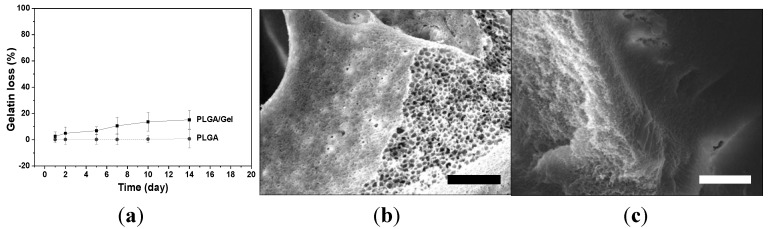
Gelatin leaching kinetic of coated scaffold (**a**); SEM microimages of PLGA scaffold (**b**); and PLGA scaffold with gelatin coating (**c**). Scale bar = 30 μm.

The stability of the gelatin coating was further evaluated by calculating the gelatin loss with time. As shown in Figure 4a, gelatin was slowly released with a cumulative release of <20% within two weeks, which was much lower than the reported cumulative loss of 30% for the gelatin-coated traditional scaffolds [20]. The SEM microimages of the PLGA and PLGA/Gel scaffolds after incubation for two weeks at 37 °C are shown in Figure 4b,c. The microstructure of the PLGA scaffold did not change after the two-week incubation and exhibited many channels on the surface. The gelatin layer in the PLGA/Gel scaffold remained tightly coated on the PLGA substrate, which corresponded to the small amounts of gelatin loss. These results indicated that the gelatin coating was stable on the surface of scaffold due to the loosened skeletal scaffold structure.

#### 2.1.2. Mechanical Property Analyses

**Figure 5:**
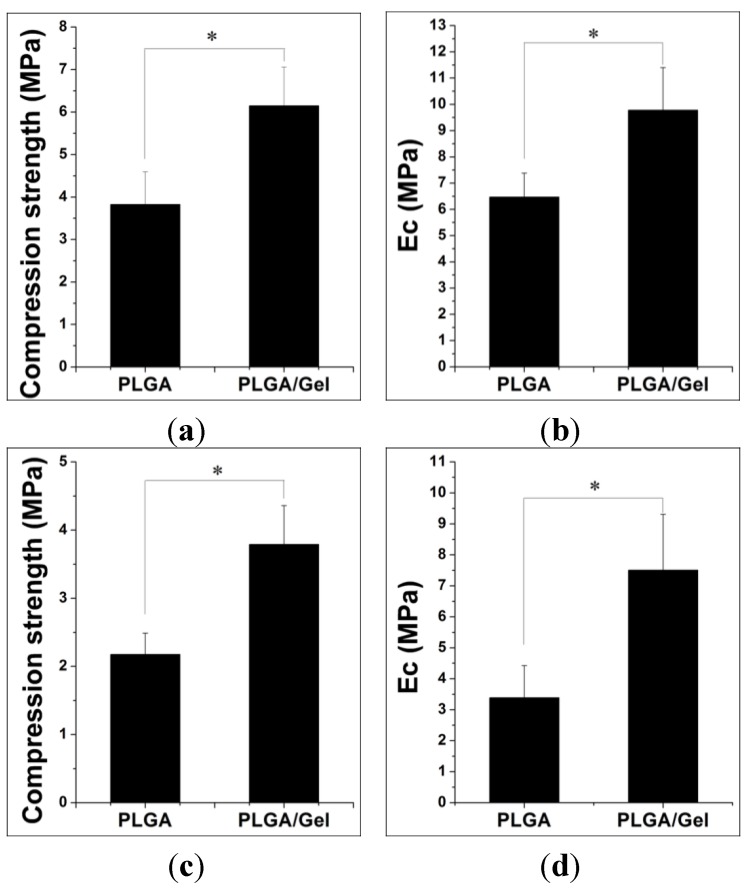
Compressive strength and Ec of PLGA and PLGA/Gel scaffolds under dry (**a**,**b**) or wet states (**c**,**d**). The data were represented as mean ± standard deviation (SD; *n* = 3; * *p* < 0.05).

The porous scaffolds are designed to provide mechanical support until the regenerative tissue or organ is structurally stabilized [28]. Therefore, the appropriate mechanical properties are crucial for such porous scaffolds. Ideally, the mechanical properties are similar to that of the supported tissue. The scaffold should maintain its structural stability and integrity in an *in vivo* biomechanical environment and provide appropriate micro-stress stimulations for the implanted cells [29]. However, the compression strength of a PLGA scaffold was found to be lower than that of a human trabecular bone (*i.e.*, 9.3 ± 4.5 MPa) [30]. A previous study demonstrated that the gelatin entrapment in the scaffold improved the compressive strength of pure polymeric scaffolds [20]. Therefore, a higher compressive strength was expected after the gelatin modifications in our study. As depicted in Figure 5, the compressive strengths and elastic moduli (Ec) of the scaffolds were indeed increased. For dry samples, the compressive strength and Ec of the PLGA/Gel scaffold reached 6.15 ± 0.91 and 9.78 ± 1.62 MPa, respectively, compared with those of 3.83 ± 0.77 and 6.47 ± 0.91 MPa for the PLGA scaffold (Figure 5a,b). The mechanical properties measured in the dry state were different than those under physiological conditions, *i.e.*, in tissue fluid at 37 °C, because of the different media. As shown in Figure 5c,d, for wet-state samples, the compressive strength and Ec of the PLGA/Gel scaffold were significantly greater than those of the PLGA scaffold (*p* < 0.05 for both). Overall, the gelatin modifications improved the mechanical properties of the PLGA scaffold for clinical use.

#### 2.1.3. Hydrophilicity Assessments

**Figure 6:**
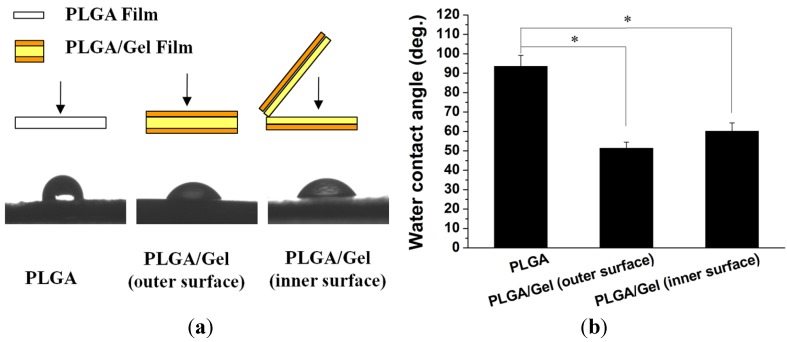
Test methods (**a**) and results (**a**,**b**) of water contact angles on outer and inner surfaces of PLGA and PLGA/Gel scaffolds. The data were represented as mean ± standard deviation (SD; *n* = 3; * *p* < 0.05).

To evaluate whether the hydrophilicity of the PLGA scaffold was improved by the addition of gelatin, the water contact angles on the outer and inner surfaces were measured for the PLGA and PLGA/Gel scaffolds (Figure 6). The water contact angle of the control PLGA scaffold outer surface was significantly greater at (93.5 ± 5.7)° compared with that of the PLGA/Gel scaffold, which decreased to (51.3 ± 3.2)° (*p* < 0.05). The contact angle of the inner surface of the PLGA/Gel scaffold (*i.e.*, (60.1 ± 4.2)°) was also significantly smaller than that of the PLGA scaffold (*p* < 0.05). These observations were expected because gelatin is a more hydrophilic molecule than PLGA; thus, the decreased outer surface contact angle indicated that the gelatin molecule was successfully incorporated onto the surface of PLGA scaffold. The decreased inner surface contact angle suggested that gelatin also diffused into the substrate microvoids.

### 2.2. Release Kinetics of rhBMP-2

**Figure 7:**
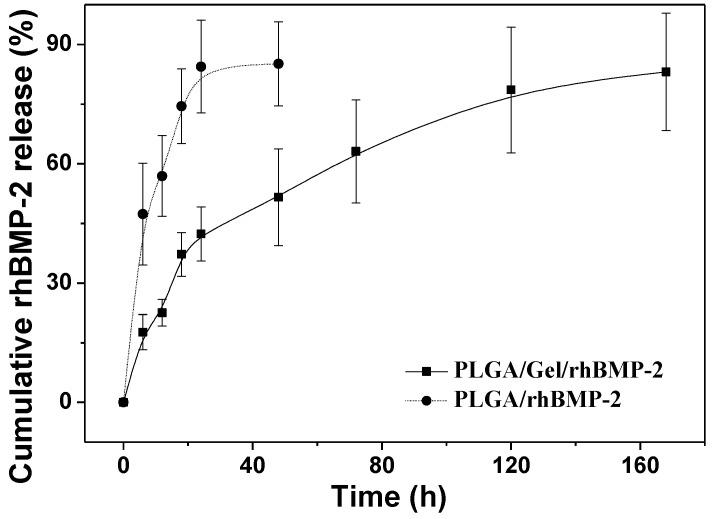
Release kinetics of rhBMP-2 from PLGA/rhBMP-2 and PLGA/Gel/rhBMP-2 scaffolds.

As shown in Figure 7, the *in vitro* cumulative release behaviors of rhBMP-2 from the composites were characterized by the percentage release of rhBMP-2 as a function of time. Without gelatin modification, *i.e.*, PLGA/rhBMP-2, a high burst release was noted; more than 80% of the loaded rhBMP-2 was released within the first 24 h. Gelatin has been extensively used in protein delivery applications. As depicted in Figure 7, rhBMP-2 showed a sustained release behavior in the PLGA/Gel/rhBMP-2 scaffold. In detail, the gelatin-coated scaffold loaded with rhBMP-2 showed a slight initial burst release within 24 h, but it was followed by a slower release over seven days.

BMPs have been shown to induce bone formation by inducing chondroblastic and osteoblastic differentiations of BMSCs [31]. BMP-2 has been shown to be the most effective agent, by which to induce complete bone morphogenesis [29], and has been approved by FDA for clinical applications [32]. It is evident that the efficacy of BMP-2 in bone tissue formation is dependent on the administered dose and the delivery mode. The sustained release has been demonstrated to be sufficient for inducing bone regeneration [33]. Yamamoto *et al.* [34] demonstrated that BMP-2 showed a very serious initial burst release within 40 min of administration from glutaraldehyde-cross-linked gelatin microspheres, followed by a sustained release. In our study, the sustained release profile of the PLGA/Gel/rhBMP-2 scaffold was different, likely due to the PLGA substrate exerting some influence on the release mechanism.

### 2.3. Cell Adhesion, Proliferation, and Differentiation in Scaffolds

**Figure 8:**
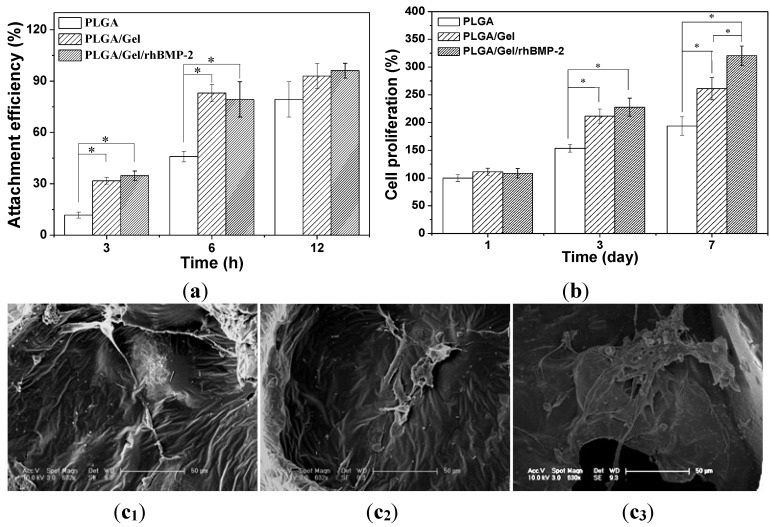
*In vitro* growth of BMSCs in scaffolds. Cell attachment efficiency on PLGA, PLGA/Gel, and PLGA/Gel/rhBMP-2 scaffolds at 3 and 6 h (**a**); Cell proliferation on PLGA, PLGA/Gel, and PLGA/Gel/rhBMP-2 on Day 1, 3, and 7 (**b**); and SEM microimages of cell attachment on PLGA (**c_1_**), PLGA/Gel (**c_2_**), and PLGA/Gel/rhBMP-2 (**c_3_**) on Day 7 (**c**). The data were represented as mean ± standard deviation (SD; *n* = 3; * *p* < 0.05).

The attachment efficiencies of BMSCs cultured on various scaffolds for 3, 6, and 12 h are summarized in Figure 8a. After culture for 3 h, the cell attachment efficiencies on the PLGA/Gel and PLGA/Gel/rhBMP-2 scaffolds reached more than 30%, which was significantly greater than the percentage on the PLGA scaffold (*i.e.*, 11.8%). When cultured for 6 h, the cell attachment efficiencies on the PLGA/Gel and PLGA/Gel/rhBMP-2 scaffolds increased to approximately 80%, greater than the 45.9% reported for the PLGA scaffold. It was believed that the modification of a PLGA scaffold with gelatin increased direct cell-material binding by increasing the surface hydrophilicity, thus, facilitating an increase in early cell adhesion. The BMSC proliferation was quantitatively monitored using the MTT assay to measure the metabolic activity of the total population of cells for one, three, and seven days. As shown in Figure 8b,c, each scaffold supported the proliferation of BMSCs within seven days. The PLGA/Gel/rhBMP-2 scaffold exhibited the highest cellular activity, whereas the PLGA scaffold showed the lowest at three and seven days, suggesting that the gelatin and rhBMP-2 modifications promoted the cell attachment and proliferation. It has been reported that the expression of integrin β1, which is promoted by rhBMP-2 [35], is required for cell spreading, adhesion, and proliferation [36,37].

**Figure 9:**
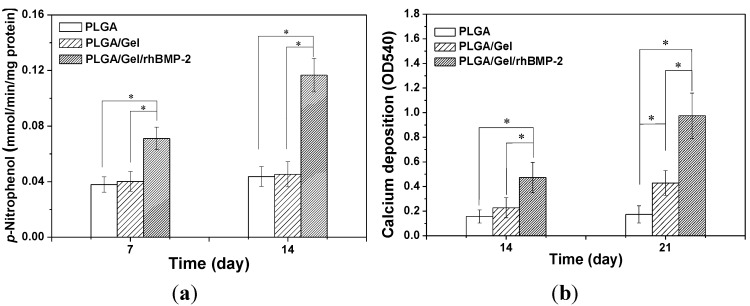
ALP activities of BMSCs in PLGA, PLGA/Gel, and PLGA/Gel/rhBMP-2 scaffolds during 14-day *in vitro* culture (**a**); Calcium deposition after culturing in PLGA, PLGA/Gel, and PLGA/Gel/rhBMP-2 scaffolds for 14 and 21 days (**b**). The data were represented as mean ± standard deviation (SD; *n* = 3; * *p* < 0.05).

**Figure 10:**
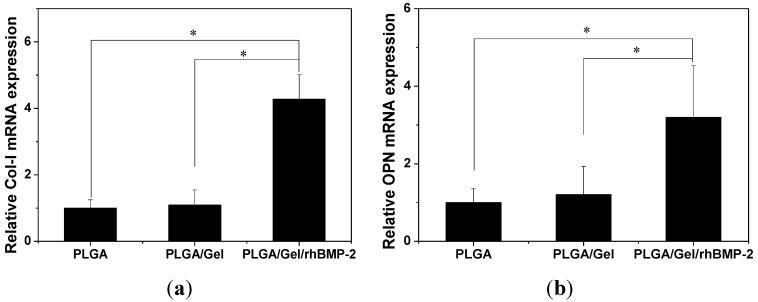
Quantitative analyses of osteogenesis-related gene expressions (*i.e.*, Col-I (**a**) and OPN (**b**)). The data were represented as mean ± standard deviation (SD; *n* = 3; * *p* < 0.05).

The cell-biomaterial interactions have been demonstrated to exert a considerable influence on the differentiation and function of BMSCs [38]. To investigate the osteogenic differentiation of BMSCs on different scaffolds, alkaline phosphatase (ALP) activity and calcium deposition were measured. ALP is a membrane enzyme commonly recognized as a marker of osteoblastic differentiation. Figure 9a shows the ALP activities of the BMSCs cultured on the different scaffolds after seven and 14 days. The significantly higher ALP activity was detected in cells cultured on the PLGA/Gel/rhBMP-2 scaffold than those on the PLGA and PLGA/Gel scaffolds (*p* < 0.05 for both). Calcium deposition was measured by alizarin red staining. As shown in Figure 9b, the quantification of ARS indicated that the deposition of calcium minerals in the PLGA/Gel/rhBMP-2 scaffold was significant higher than in the other scaffolds (*p* < 0.05). Moreover, after culturing for 21 days, the deposition of calcium mineral in the PLGA/Gel scaffold was greater than that in the PLGA scaffold (*p* < 0.05). These results demonstrated that the PLGA/Gel/rhBMP-2 and PLGA/Gel scaffolds promoted the osteogenic differentiation of BMSCs. The quantitative analyses of the expressions of the osteogenesis-related genes (*i.e.*, collagen-I (COL-I) and osteopontin (OPN)) were performed by quantitative reverse transcription-polymerase chain reaction (qRT-PCR) (Figure 10), which supported the results observed from the calcium deposition measurements. The expression levels of COL-I and OPN were higher in the PLGA/Gel/rhBMP-2 scaffold than those in the PLGA/Gel and PLGA scaffolds. This result was attributed to the gelatin coating and the gelatin-immobilized rhBMP-2. All above data indicated that the bioactivity of rhBMP-2 was well retained during the surface-coating process, and the sustained release of rhBMP-2 showed improved BMSC differentiation.

## 3. Experimental Section

### 3.1. PLGA Scaffold Fabrication

A PLGA scaffold was fabricated *via* combining phase separation and particulate leaching. Two grams of PLGA (LA:GA = 50:50, 14,000 kDa, Jinan Daigang Biomaterial Co., Ltd., Jinan, China) was added into 10.0 mL of NMP (Sigma-Aldrich Co., Shanghai, China). The mixture was continuously stirred until the polymer was thoroughly dissolved. Next, the sieved sodium chloride particulates of 100–300 μm in diameter were added into the PLGA solution. The weight ratio of the salt particulates to the polymer composites was 6:1. The mixture was cast in a homemade glass cylinder with a removable bottom. To remove NMP and salt particulates, the bottom was removed, and the mixture was immersed in distilled water for 5 days with the water exchanged every 6 h. Subsequently, any water remaining in the scaffold was exchanged by ethanol. Finally, the PLGA scaffold was obtained after 3 days of lyophilization.

### 3.2. PLGA/Gelatin (PLGA/Gel) and PLGA/Gel/rhBMP-2 Hybrid Scaffold Fabrication

First, 0.01 g/mL of gelatin solution was prepared by dissolving gelatin (Sigma-Aldrich Co., Shanghai, China) in distilled water at 37 °C. rhBMP-2 was supplemented to a final concentration of 5.0 μg/mL. Subsequently, the PLGA scaffold was immersed in the gelatin solution or the gelatin/rhBMP-2 solution at 37 °C under vacuum for 30 min before fumigation by glutaraldehyde at 4 °C for 24 h to complete the cross-linking reaction. To clear any residual glutaraldehyde in the scaffold, the hybrid composite was vacuum-drawn and washed with ethanol several times. Finally, the PLGA/Gel and PLGA/Gel/rhBMP-2 scaffolds were lyophilized for 3 days and stored at 4 °C in a desiccator until use.

### 3.3. Characterization of Scaffolds

The porosities of the scaffolds were determined using the ethanol replacement method [21]. The microstructures of the scaffolds were examined by scanning electron microscopy (SEM, Philips XL30, Eindhoven, The Netherlands). The scaffolds were fractured after snap-freezing, sputter-coated with gold, and observed at an accelerating voltage of 15 kV. To assess the gelatin distributions in the scaffolds, gelatin labeled with fluorescein isothiocyanate (FITC, Aladdin Reagent Co., Ltd., Shanghai, China) was used. The horizontal sections (100 μm thick) of the scaffolds were observed under a fluorescence microscope (TE200-U, Nikon Instruments Inc., Tokyo, Japan). The gelatin contents in the scaffolds were determined based on the change in dry weight of the scaffolds before and after modification. Cylindrical samples of 5 mm in diameter and 10 mm in height were chosen for mechanical strength tests using a universal testing machine (Instron 1121, High Wycombe, UK). The compressive strengths were measured at a crosshead speed of 2.0 mm/min. Three replicates were tested for each condition (*n* = 3).

The weight loss of the gelatin in the scaffolds was determined at 37 °C under agitation at 60 rpm over a period of 2 weeks. At predetermined time intervals, the scaffolds were freeze-dried and weighed. The percentage of gelatin loss was calculated as Gelatin loss = (W_1_ − W_2_)/(W_1_ − W_0_) × 100%, wherein W_0_ is the dry weight of the original surface-coated scaffolds, W_1_ is the dry weight of the modified scaffolds at baseline, and W_2_ is the dry weight of the surface-coated scaffolds at time *t*. In addition, the scaffolds were examined by SEM after 2 weeks of incubation.

The PLGA and PLGA/Gel films were prepared for static air-water contact angle measurements using the sessile drop method on a contact angle system (VCA 2000, AST, Bellerica, MA, USA). A solution of 20% PLGA was poured in a glass plate and then immersed in double distilled water (ddH_2_O) for 24 h. After exchanging NMP by water, the PLGA film was obtained. The PLGA/Gel film was prepared by exposing PLGA film to a 1% gelatin solution for 24 h. Furthermore, the PLGA/Gel film was sliced in the middle, and the hydrophilicities of the PLGA and PLGA/Gel films (outer surface and inner surface) were detected after lyophilization.

### 3.4. In Vitro Release Study

The PLGA scaffold was immersed in 5.0 μg/mL rhBMP-2 solution to obtain PLGA/rhBMP-2. The PLGA/Gel/rhBMP-2 and PLGA/rhBMP-2 scaffolds were used for the *in vitro* release study. Each scaffold (5 × 5 × 5 mm^3^) incorporating rhBMP-2 was incubated in 5.0 mL of phosphate-buffered saline (PBS) at 37 °C under stirring at 60 rpm. At specified time intervals, 0.2 mL of the supernatant was collected, and an equal volume of fresh PBS was added. The content of rhBMP-2 was measured using an enzyme-linked immunosorbent assay (ELISA) kit (R&D System) according to the manufacturer’s instruction. The release profiles were obtained by plotting the percentage of cumulatively content of released rhBMP-2 against time. The experiments were performed in triplicate.

### 3.5. Cell Adhesion, Proliferation, and Differentiation Assays

#### 3.5.1. Bone Mesenchymal Stem Cell (BMSC) Isolation

Two-month-old New Zealand white rabbits were sacrificed for BMSC isolation. Bone marrow aspirate (5.0 mL) were obtained from the rabbit tibias and subsequently cultured. Briefly, the isolated cell pellets were resuspended in 5.0 mL of the complete Dulbecco’s Modified Eagle’s Medium (DMEM; Gibco BRL, Grand Island, NJ, USA) supplemented with 10% (v/v) fetal calf serum (FCS; Gibco BRL, Grand Island, NJ, USA) and 100 IU/mL penicillin-streptomycin (Sigma, Shanghai, China). The cells were seeded in culture dishes (Corning Costar Co., Cambridge, MA, USA) and cultured at 37 °C in an incubator with 5% carbon dioxide (CO_2_). Non-adherent cells were discarded when the medium was changed after 24 h. Subsequently, the medium was replaced every other day until the cells reached 80% confluence. Then, the cells were washed with PBS, digested by 0.25% trypsin/ethylenediamine tetraacetic acid (trypsin/EDTA; Sigma, Shanghai, China), and subcultured at a 1:3 dilution under the same condition until the third passage.

#### 3.5.2. Cell Adhesion and Proliferation Assays

For the adhesion and proliferation studies, 1 × 10^5^ BMSCs were seeded on the scaffolds (5 × 5 × 3 mm^3^). The adhesion and proliferation of the cells for each sample were determined by a standard 3-(4,5-dimethylthiazoyl-2-yl)-2,5-diphenyltetrazolium bromide (MTT) assay. At 6 and 12 h post-seeding, the culture medium was discarded, and the unattached cells were washed away with PBS. The attached cells on the scaffolds were detached by trypsin/EDTA, and the number of cells was carefully counted. Finally, the cell attachment efficiency was calculated according to the following equation: Cell attachment efficiency = N_1_/N_0_, where N_1_ and N_0_ were the numbers of attached and seeded cells, respectively.

Cell proliferation was determined on Day 1, 3, and 7. The scaffolds were incubated in an MTT solution (5.0 mg/mL in PBS) for 4 h. After the removal of the MTT solution, the acidified isopropanol (0.2 mL of 0.04 M HCl in 10 mL of isopropanol) was added to solubilize the resultant formazan product. The absorbance of the extractant at 492 nm was recorded on a Thermo Electron MK3 spectrophotometer (Thermo Scientific, Hudson, NH, USA). The relative cell number (%) was determined by comparing the absorbance to that for PLGA on Day 1. The mean value of nine readings for each sample was used as the final result. The cell proliferation on Day 7 was also observed by SEM.

#### 3.5.3. Cell Differentiation Assays

Alkaline phosphatase (ALP) activity was determined after culturing the cells in DMEM/F12, FBS (10%, V/V) for 7 and 14 days. Briefly, the medium of each well was carefully removed. Then, the cells were washed three times with PBS, lysed in radioimmunoprecipitation assay (RIPA) buffer, frozen at −80 °C for 30 min, and thawed at 37 °C. Then, *p*-nitrophenol phosphate substrate (*p*NPP) solution was added, and the samples were incubated in the dark for 30 min at 37 °C. The reaction was terminated with 3.0 M NaOH, and the ALP activity was read on a multifunction microplate scanner (Tecan Infinite M200) at 405 nm. Measurements were compared with p-nitrophenol standards and normalized by the total protein content, which was determined with a bicinchoninic acid (BCA) kit (Pierce Biotechnologies, Rockford, IL, USA).

Calcium deposition was determined by alizarin red S (ARS) staining of the BMSCs after culture in DMEM/F12, FBS (10%) for 14 and 21 days. After three 5 min rinses in water, the scaffolds were incubated in ARS stain solution (0.1% ARS in Tris-HCl buffer, pH 8.0, Sigma, Shanghai, China) for 30 min at 37 °C. The scaffolds were then washed in distilled water three times for 5 min each. The stained samples were treated with 10% (w/v) cetylpyridinium chloride in 10.0 mM sodium phosphate for 15 min at room temperature. The absorbance of ARS at 540 nm was recorded on a Thermo Electron MK3 spectrophotometer.

The osteogenesis-related gene expression levels were quantitatively assessed using RT-qPCR for BMSCs cultured on various scaffolds incubated for 14 days. Total RNA was extracted using TRIzol Reagent (Invitrogen) according to the manufacturer’s protocol. The total RNA concentration and purity were estimated using Nanodrop Plates (Tecan Infinite M200, Tecan Group Ltd., Maennedorf, Switzerland), and the RNA was reverse transcribed as described in the M-MLV manual (Promega). RNA was added to a 20.0 μL reverse transcription reaction mixture containing 5 × M-MLV buffer, dNTP mixture, RNase inhibitor, RTase M-MLV, RNase free dH_2_O, and oligo (dT) primer. The expression levels of osteogenic markers were quantified using a qPCR SYBR Green Mix Kit (Stratagene). The primer sequences specific for the target gene and the internal control gene (glyceraldehyde-3-phosphate dehydro-genase (GAPDH)) used for qRT-PCR are listed in Table 2. The specificities of the listed oligonucleotides were checked by Basic Local Alignment Search Tool (BLAST) against the rabbit RefSeq RNA database at NCBI. The qPCR amplification was performed as follows: initial heating at 95 °C for 10 min, followed by 40 cycles at 95 °C for 30 s, 58 °C for 60 s, and 72 °C for 60 s. The expression levels were determined using threshold cycles (Ct) that were determined by the iCycler iQ Detection System software. The relative transcript quantities were calculated using the ΔΔCt method. The GAPDH gene was used as a reference gene and was amplified along with the target genes from the same cDNA samples. The difference in the Ct of the sample mRNA relative to the GAPDH mRNA was defined as the ΔCt. The difference between the ΔCt of the control cells and the ΔCt of the cells grown on the substrates was defined as the ΔΔCt. The fold change in mRNA expression was expressed as 2^−ΔΔCt^.

**Table 2:** Sequences of primers for quantitative reverse transcription-polymerase chain reaction (qRT-PCR).

Gene	Forward Primer Sequence	Reverse Primer Sequence
COL-I	5′-CTCGCTCACCACCTTCTC-3′	5′-TAACCACTGCTCCACTCTG-3′
OPN	5′-CGTGGATGATATTGATGAGGATG-3′	5′-TCGTCGGAGTGGTGAGAG-3′
GAPDH	5′-GATGGTGAAGGTCGGAGTG-3′	5′-TGTAGTGGAGGTCAATGAATGG-3′

### 3.6. Statistical Analysis

The data were presented as mean ± standard deviation (SD). The independent and replicated experiments were used to analyze the statistical variability of the data analyzed using Student’s *t*-test, and *p* < 0.05 was considered to be significant.

## 4. Conclusions

In this study, a gelatin tight-coated rhBMP-2-incorporated PLGA-based scaffold with a loosened skeleton was fabricated. Because of the special structure, the gelatin molecule easily diffused throughout the scaffold. By cross-linking with glutaraldehyde, the gelatin coating was tightly bound with both the internal and external surfaces of microscale channel. The modification with gelatin also significantly improved the mechanical strength and hydrophilicity of these surfaces. For gelatin-coated scaffolds with rhBMP-2, a sustained release behavior was observed *in vitro*, which enhanced the attachment, proliferation, and differentiation of BMSCs. The obtained data collectively demonstrate that the gelatin-coated PLGA scaffolds can effectively deliver bioactive factors and hold great promise for bone tissue engineering.
